# Exogenous features versus prior experiences modulate different subregions of the right IPL during episodic memory retrieval

**DOI:** 10.1038/srep11248

**Published:** 2015-06-09

**Authors:** Sze Chai Kwok, Emiliano Macaluso

**Affiliations:** 1 Key Laboratory of Brain Functional Genomics, Ministry of Education, Shanghai Key Laboratory of Brain Functional Genomics, Institute of Cognitive Neuroscience, School of Psychology and Cognitive Science, East China Normal University, Shanghai, China; 2 NYU-ECNU Institute of Brain and Cognitive Science, NYU-Shanghai University, Shanghai, China; 3 Neuroimaging Laboratory, Fondazione Santa Lucia, Istituto di Ricovero e Cura a Carattere Scientifico (IRCCS), Rome, Italy

## Abstract

The fractionation view holds that distinct cognitive operations are mediated by subregions of the inferior parietal lobule (IPL). Within IPL, we hypothesised that retrieval-related activity in different parts of the right supramarginal gyrus (rSMG) may be modulated differentially by information acquired via different combinations of attention signals at encoding. We had two groups of participants watch a 42-min TV episode and, after a 24-hr delay, perform a temporal-order judgment task during fMRI. Each retrieval trial comprised three images presented sequentially, requiring participants to judge the temporal order between the first and last images while ignoring the second image (“distractor”). We manipulated the bottom-up factor by presenting distractors that were extracted from either an event-boundary or a non-boundary of the movie. The top-down factor was manipulated by instructing one group perform a segmentation task reporting the event-boundaries at encoding, while the other group watched the movie passively. Across groups, we found that the stimulus-related factor modulated retrieval activation in the anterior rSMG (areas PFt and PFop), whereas the goal-related influence of prior segmentation interacted with this effect in the middle rSMG (area PF), demonstrating IPL segregation during retrieval as a function of prior bottom-up vs. top-down attention signals.

The human inferior parietal lobule (IPL) is a structurally and functionally heterogeneous part of the cortex^1^,^2^. Recent diffusion-weighted imaging^3^,^4^ and resting-state connectivity studies^5^,^6^,^7^ showed that IPL subregions go beyond the traditional areal subdivision of rostal BA 40 and caudal BA 39. High resolution anatomical studies using an observer-independent definition of cytoarchitectonic borders revealed that the supramarginal gyrus (SMG, BA40) can be divided into five subregions (PFop, PFt, PF, PFm, and PFcm) and the angular gyrus (AG, BA39) into two subregions (PGa and PGp)^8^,^9^,^10^. Using functional clustering techniques, the IPL was subdivided into seven clusters that are arranged hierarchically according to their connectivity to the whole brain^11^. Moreover, with analyses integrating resting-state and task-related MRI data, Nelson *et al.* provided a detailed parcellation for the left IPL^12^. Altogether, these demonstrations of anatomo-functional differences within the IPL prompted some authors to put forward a “fractionation” view, which holds that distinct cognitive operations are mediated by anatomically segregated and functionally specialised subregions within the IPL^12^,^13^,^14^.

By this view, the well-delineated subregions in the IPL would be recruited differentially according to task demands and the processes implicated. In the left hemisphere, one topic of increasing interest regards memory retrieval. The left IPL has featured frequently as a region that contains signatures of memory traces^15^. The six sub-modules delineated in Nelson *et al.*^12^ are implicated in different “retrieval success” effects^16^ and support the hypothesis that divisions within the left IPL provide functionally distinct contributions towards recognition memory^14^. In the same hemisphere, another example that illustrates sub-regional specificity concerns the responses of the anterior IPL to the observation of tool versus hand actions^17^. Only one subregion (out of eleven) within the anterior SMG was specifically associated with tool-action observation^18^. Thus, studies that made use of high anatomical precision revealed functional specificity of subregions of the left IPL, in line with the cytoarchitectonic probabilistic maps^9^,^10^.

Anatomo-functional fractionation for the IPL has also been noted in the right hemisphere^19^,^20^. The involvement of the anterior part of the right SMG in detection related processes in perception^21^,^22^ and in memory^23^ led to the suggestion that the rSMG may subserve bottom-up attention processes, including signals associated with the retrieval of mnemonic representations. According to the Bottom-Up Attention (BUA) hypothesis^24^,^25^, activation in the anterior SMG during memory retrieval can be attributed to the process in which recollected episodic details capture bottom-up attention. Functional connectivity analyses revealed that this area is connected with the medial temporal lobe (MTL)^23^, further supporting the attentional role of the rSMG in memory retrieval. In line with the view concerning the detection related role of the right IPL in orienting attention to salient sensory input^22^, the BUA hypothesis posits that the rSMG may be engaged, not only by internal memories attracting attention during retrieval, but also by salient external cues that trigger retrieval operations^24^. We have accordingly addressed this interplay between memory-driven attention and memory retrieval using an interference paradigm that engaged both attention and memory systems concurrently^26^. In that study, following the encoding of a 42-min movie and a retention period, participants were asked to judge the temporal order of two probe images extracted from the movie. These two images were presented sequentially, but were interleaved by an additional task-irrelevant image (also from the movie) which served as an attention distractor. We showed that the rSMG was sensitive to the distractor-probes temporal relationship in the movie. In the framework of bottom-up attention capture, we suggested that the task irrelevant distractors acted as “automatic”, attention-grabbing retrieval cues, as they belonged to the same memory representation as the task-relevant probe images.

Based on the probabilistic maps^9^,^10^ and transmitter receptor-based architectonics^8^, the regions obtained in Kwok *et al.*^26^ appear to be in the anterior and middle SMG, respectively. The anterior and middle SMG have different patterns of connectivity^3^,^4^ and have been associated with different functions^11^. The anterior SMG is connected with sensory-motor regions around the central sulcus, as well as the superior temporal gyrus, putamen, and insula areas that respond to targets in oddball tasks^27^, indicating its possible role in bottom-up, stimulus-related attention control. By contrast, the middle SMG connects with the superior parietal lobule and the lateral prefrontal cortex, which are key areas for top-down control^28^,^29^. The existence of top-down endogenous signaling within the SMG goes beyond the traditional view linking the ventral parietal cortex with stimulus-driven attention^30^, and fits with the emerging idea that the ventral system integrates stimulus-driven information with endogenous signals related to expectation and goals, see^21^,^31^,^32^ for reviews.

Given the likelihood of attention operating within memory representations, the different contributions of bottom-up and top-down factors in the rSMG, and the detailed sub-regional architecture of the rSMG, presently we sought to elucidate how information acquired via different combinations of attention signals at encoding modulates brain activity at retrieval, when the same information was presented again – in a “bottom-up” manner – as task-irrelevant distractors. We employed a modified version of our previous paradigm to investigate the interplay between attention and memory during the retrieval of temporal information about naturalistic material^26^. We presented task-irrelevant distractors during retrieval of the temporal order of two memory probe images, but now further manipulated the amount of bottom-up and top-down attentional resources allocated to these distractor stimuli during encoding. Top-down signals refer to signals arising from some task/goal-related instructions given to the subject (i.e., the source of the signal is not present in the sensory input), whereas bottom-up signals refer to information that is physically present in the input, irrespective to whatever task or goal has been given to the subject.

We manipulated bottom-up attention at encoding using event boundaries^33^. According to event-segmentation theory, event models are fluid representations that incorporate the perceptual details of the current experience with semantic knowledge of similar past experiences^34^. When perceptual details become incongruent with the model, the event model is updated to accommodate these changes. The points at which these updates occur are called event boundaries^35^. Event boundaries help people remember the happenings^36^, leading to better long-term memory for boundary vs. non-boundary information^37^. Event boundaries are detected based on changes of external/sensory information, and in the current study, were used to index the bottom-up contribution to the encoding of information to memory (i.e., the factor of “*Boundary*”, with distractor images extracted either from “boundary” or “non-boundary” regions of the movie). In contrast, the level of top-down attention dedicated to the processing of the distractor images during movie encoding was manipulated by incorporating an explicit event segmentation task during movie-viewing. We hypothesised that the two types of attention signals at encoding would influence the mnemonic statuses of these distractor images differently within the memory engram, in turn leading to differential effects when these images were (re-)presented as task-irrelevant distractors during retrieval. The processing of the stimulus related aspects of the boundary events should be common for the two groups, whereas the top-down, goal-related effect of having explicitly detected and reported these events should be specific for the segmentation group.

On day 1, all participants were presented with a 42-min TV series episode. They were however divided into two groups that received two different sets of instructions regarding the encoding of the episode (factor of “*Group*”: segmentation / viewing). Participants in the “viewing group” were told to watch the movie passively (as of^26^; i.e., no top-down goal during encoding), whereas the “segmentation group” was instructed to perform an event segmentation task to detect and report any event transition in the movie (see Segmentation task, Methods). The following day (with 24-hr retention delay), all participants performed an identical temporal order judgment task during fMRI. Each retrieval trial comprised three images presented sequentially, and the task was to judge the temporal order of the first and the last images (memory probes, “Im1/Im2”) while ignoring the second image that was task-irrelevant (distractor, “D”; Fig. 1B). Within-subjects, we manipulated whether the distractor frame was extracted from a boundary or not (“*Boundary*”: boundary/non-boundary), plus the temporal distance between the memory probes (“*Distance*”: short/long) that we previously found to interact with the effect of the task-irrelevant distractors^26^. This paradigm allowed us to study the effect of a given experimental manipulation on memory retrieval (i.e., top-down vs. bottom-up signals), without making these specific aspects of the stimuli to be directly relevant for the participants. Although the distractors (particularly boundary distractors) might provide a search “description” for the memory retrieval^38^, such description does not necessarily provide direct information about the relative before/after occurrence of the Im1 and Im2 memory probes, which was the task that participants were asked to perform.

In sum, we manipulated top-down and bottom-up signals at encoding and assessed how these manipulations impacted on subsequent retrieval by comparing trials including different types of distractor images. The main analyses focused on the distractor-type (boundary vs. non-boundary) and its interaction with the temporal distance between the memory probes (short vs. long), as well as on any modulation of these according to the task-relevance of the boundary events during encoding (“*Distance × Boundary × Group*” interaction). The memory task required chronological order judgement of events and, prompted by what was previously demonstrated^23^,^26^, we anticipated that the critical attention effects (i.e., effect of “*Boundary*” and/or interaction between “*Boundary*” and “*Group*”) would be dependent on the temporal distance between the two task-relevant memory probes (factor of “*Distance*”). Supported by the anatomical knowledge^8^,^10^, and motivated by recent functional insights into the attention-in-memory phenomenon^23^,^26^, our investigation combined the functional findings with the existing cytoarchitectonic knowledge by targeting relevant voxels within the five subregions of the right SMG (PFop, PFt, PF, PFm, and PFcm) as defined by Casper *et al.*^9^. We chose to utilise the Jülich–Düsseldorf atlas^8^,^9^,^10^ because this is the most widely-used atlas regarding the IPL cytoarchitecture^39^, particularly in relation to mechanisms of attention control^40^. We predicted an influence of the manipulation of bottom-up attention at encoding to affect distractors processing in the rSMG at retrieval, and that the level of top-down effort exerted during encoding would further modulate this effect, with a possible segregation of these bottom-up and top-down influences in different subregions of the rSMG.

## Results

The paradigm was a 2 × 2 × 2 mixed factorial design, with “*Distance*” (short / long: temporal distance between memory probes) and “*Boundary*” (D_BORDER_ / D_non-BORDER_: distractor frames at event boundary, or not) as the within-subjects factors, and “*Group*” (segmentation / viewing) as the between-subjects factor.

### Retrieval times (RT) and accuracy analysis

**Figure 1:**
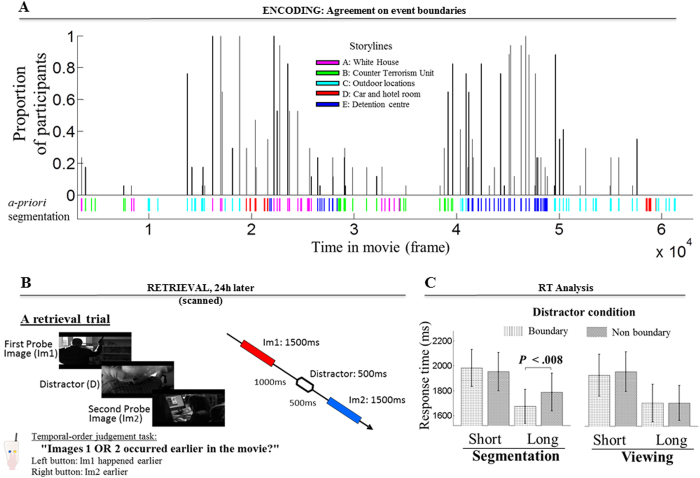
Segmentation task results, the retrieval paradigm, and retrieval times results. **(A)** Individuals agree about the locations of event boundaries. The plot shows the proportion of participants who identified event boundaries within 2-s intervals with respect to the 141 boundaries coded by our *a-priori segmentation*. The coloured bars in the bottom part show the onset of epochs, as coded by our *a-priori segmentation*, for the five concurrent storylines portraying different characters at various locations in the 42-min movie (storyline A: in the White House; B: in a Counter Terrorism Unit; C: various outdoor locations; D: in a car and then in a hotel room; E: in a detention centre). **(B)** The retrieval test was performed after a 24-hour delay during fMRI. Each retrieval trial consisted of a triplet of images: First Probe Image (“Im1”, for 1500 ms), Distractor Image (“D”, for 500 ms), and Second Probe Image (“Im2”, for 1500 ms), which were presented sequentially. The Im1 and D images were separated by a 1000 ms blank screen, whereas D and Im2 separated by a 500 ms blank screen. The task required the participant to judge the temporal order of the two probe images while ignoring the distractor image. Participants were instructed to respond with a keypress with their right hand as soon as they made a decision once the second probe image appeared. The distractor was either a boundary or a non-boundary image. The three images illustrated here are stills taken from the American TV series “*24*” (produced by The Fox Broadcasting Company). **(C)** Mean retrieval times by conditions for the two groups (active segmentation and passive viewing). Boundary distractors reduced RT in long distance trials, with such behavioural advantage being stronger in the segmentation group than in the viewing group. Error bars denote standard error (s.e.m.).

A mixed-design repeated-measures ANOVA on RT revealed the expected effect of temporal distance between the two probe images (main effect of “*Distance*”, *F* (1, 32) = 119.52, *P* < 0.001; slower RTs in short compared to long trials, see^26^,^41^), and interactions between the factors of “*Distance*”, “*Boundary*” and “*Group*”. Specifically, we found a 2-way “*Distance × Boundary*” interaction, *F* (1, 32) = 5.73, *P* = 0.023, indicating differential impact of the D_BORDER_ distractors on RT depending on the temporal distance between the memory probes. The analysis also showed a significant 3-way “*Distance × Boundary × Group*” interaction, *F* (1, 32) = 7.03, *P* = 0.012, indicating a further specificity according to the factor “*Group*”. The data displayed in Fig. 1C illustrate that this complex interaction arose primarily from the difference between trials including D_BORDER_ vs. D_non-BORDER_ distractors specifically for the long trials in the segmentation group. This was confirmed by a *t* test directly comparing D_BORDER_ vs. D_non-BORDER_ conditions that showed a significant effect only in the long trials in the segmentation group, *t* (16) = 3.04, *P* = 0.008.

The RT results demonstrated that the mnemonic statuses of the task-irrelevant distractors determined by bottom-up signals (here, the *Boundary*-factor) and top-down signals (here related to the *Group*-factor) at encoding modulated their impact on the retrieval of temporal information about complex stimulus material. The explicit task-related segmentation experiences, which emphasised the processing of the boundary images during encoding, accentuated the effects of such distractors upon subsequent retrieval. Specifically, the fastest RT was observed when the irrelevant distractor image entailed both high top-down and bottom-up signals at encoding (i.e., boundary-distractors in the segmentation group, see Fig. 1C), indicating a combined impact of both top-down and bottom-up signals on behavioural performance.

The analysis of the retrieval performance revealed significant main effects of “*Distance*”, *F* (1, 32) = 180.94, *P* < 0.001, and of “*Group*”, *F* (1, 32) = 4.28, *P* = 0.047. The effect of temporal distance (with better performance for long than short trials: 80.9% vs. 60.1%) was expected and in line with several previous studies that assessed temporal order retrieval using naturalistic images^41^,^42^. The main effect of “*Group*” was driven by marginally lower performance in the segmentation group compared with the viewing group (67.3% vs. 73.7%). A possible reason for this is that the segmentation task caused the subjects to focus their attention on the event boundaries, reducing the amount of resources dedicated to the processing of the other parts of the movie, from which the memory probes were extracted.

### Functional segregation of stimulus-driven vs. top-down influences in the rSMG

For the functional data, we asked whether the effects of different mnemonic statuses of the task-irrelevant distractors determined by bottom-up (boundary/non-boundary events) and top-down (active segmentation/passive viewing) attention could be mapped onto specific subregions of the rSMG. We examined the pattern of activation in five subregions (PFop, PFt, PF, PFm, and PFcm)^9^ using a volume-of-interest (VOI) approach.

We first investigated the role of bottom-up signals by comparing trials that included distractors extracted from “boundary vs. non-boundary” locations of the movie, that is, the main effect of “*Boundary*”. Consistent with the behavioural data, this contrast did not reveal any significant effect.

**Figure 2:**
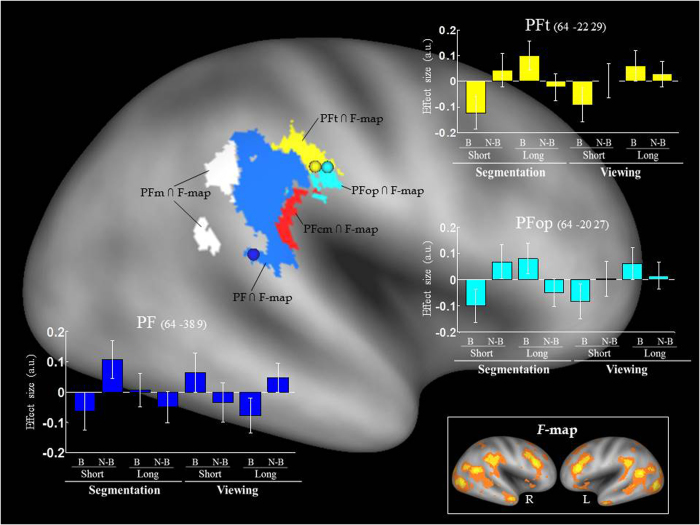
The VOI locations and signal plots by condition and group, and the *F*-map. We projected the five volumes of interest (VOI) onto an inflated surface rendering of the right cerebral cortex (http://brainvis.wustl.edu/wiki/index.php/Caret:About)^63^. The coloured patches depicted the VOIs generated by intercepting each of the five relevant cytoarchitectonic areas in the rSMG (i.e., PFop, PFt, PF, PFm, PFcm)^9^ with the functional data (*F*-test). The bottom right inset shows the results of the omnibus *F*-test, at a low threshold of *p*-unc. = 0.05 (R: right hemisphere, L: left hemisphere). The signal plots for the anterior SMG shows the “*Distance × Boundary*” interaction [(D_BORDER_ > D_non-BORDER_) _Long_ > (D_BORDER_ > D_non-BORDER_) _Short_] in the PFt (in yellow) and in the PFop (in cyan), whereas the signal plot for the middle SMG shows the 3-way interaction associated with the difference between the two groups in the PF (in blue). The probability of this latter peak belonging to the PF was estimated to be 50% by the SPM Anatomy toolbox^64^. The signal plots refer to the peak voxels (cf. spheres within the respective VOIs on the inflated surface; see also Tab. 1). Effect sizes are mean adjusted (sum to zero) and expressed in arbitrary units (a.u. ± 90% CI).

Second, we examined whether the effect of “*Boundary*” (i.e., D_BORDER_ vs. D_non-BORDER_ distractors) interacted with the factor of “*Distance*”. Given the behavioural results reported above (see Fig. 1C), we expected the D_BORDER_ distractors to be mostly effective when the distance between Im1/Im2 was long, that is, (D_BORDER_ > D_non-BORDER_) _Long_ > (D_BORDER_ > D_non-BORDER_) _Short_. The analysis confirmed this prediction and revealed significant activation in the PFt and the PFop subregions in the anterior part of the rSMG (Fig. 2, signal plots in yellow and cyan; and Table 1). In these two subregions, irrespective of group, the retrieval related activity was larger when the trial included a border than a non-border distractor, specifically in long distance trials. The reverse interaction contrast did not reveal any significant effect.

Our next main question concerned the influence of top-down task constraints that here was operationalised with the factor of “*Group*”. We hypothesised that if the top-down task priority at encoding was critical in affecting attention at retrieval, the segmentation task would impose a differential influence on the memory retrieval task, resulting in stronger activation in the segmentation group than in the viewing group. This was tested with the 3-way interaction: [(D_BORDER_ > D_non-BORDER_) _Long_ > (D_BORDER_ > D_non-BORDER_) _Short_] _SEGMENTATION_ > [(D_BORDER_ > D_non-BORDER_) _Long_ > (D_BORDER_ > D_non-BORDER_) _Short_] _VIEWING_. Among the five rSMG subregions, the only area showing a significant 3-way interaction was the PF. In this subregion, the pattern which was observed in PFt and PFop irrespective of group (i.e., [(D_BORDER_ > D_non-BORDER_) _Long_ > (D_BORDER_ > D_non-BORDER_) _Short_]) was found in the segmentation group only. Note that these group-related differences/activation pattern in PF were highlighted using “Condition × Group” interactions, which ensured that these did not reflect some uncontrolled, overall difference between the groups (see blue signal plot in Fig. 2 and Table 1).

For completeness, we performed a 4-way ANOVA that directly compared the influence of top-down and bottom-up signals in the relevant rSMG subregions. We should stress that this additional analysis made use of non-independent contrasts (i.e., 2- and 3-way interactions to identify the relevant regions, with the contrasts tested in the 4-way ANOVA), and therefore it was reported for confirmatory purposes only. The main voxel-level analyses above revealed significant “*Distance × Boundary*” interactions in the PFt and PFop, while in the PF we found a significant “*Distance × Boundary × Group*” interaction (see Fig. 2). However, this regional specificity may just reflect an effect of statistical thresholding, for example, with the 3-way interaction simply not reaching significance in the PFt/PFop (Tab. 1). To eliminate this possibility, we extracted the individual data from the peak-voxel of the three relevant areas (PF, PFt, and PFop; see Table 1, peaks reported in “bold”) separately for the 8 experimental conditions, and entered these in a mixed-design 4-way ANOVA in SPSS (Version 21.0. Armonk, NY: IBM Corp.). Within this, we tested for the “*Region* × *Distance × Boundary*” and “*Region* × *Distance × Boundary × Group*” interactions, seeking to highlight any region-specificity over and above any thresholding effect. The first interaction was significant, *F* (2, 64) = 5.50, *P* = 0.006, demonstrating that the impact of the different (boundary vs. non-boundary) distractors was indeed different in the three rSMG subregions. The further interaction with the factor of “*Group*” revealed a statistical trend, *F* (2, 64) = 2.48, *P* = 0.092, which suggested that the manipulation of top-down attention (segmentation vs. viewing) contributed to the difference between the subregions (see signal plots in Fig. 2). This additional between-regions ANOVA, albeit not independent, confirmed our main results.

It is also informative to examine the fMRI data more broadly. We performed a whole-brain analysis to test the same contrasts reported for the VOI analyses. For the “*Distance × Boundary*” 2-way interaction, the peak of maximal activation in the entire brain was found at x, y, z = 64, −20, 27 (*Z* = 3.59); that is, the same coordinates revealed in the main analyses above (area PFop). The same cluster (35 voxels, at a cluster-forming voxel-level threshold of *p*-unc. = 0.001) extended dorsally/posteriorly into the PFt region, encompassing also the peak located in the PFt (x, y, z = 64, −22, 29). No other part of the IPL was activated in the whole-brain analysis at *p*-unc. = 0.001. As for the 3-way interaction involving the group effect, again the whole-brain data showed that the reported effect in area PF (x, y, z = 64, −38, 9; *Z* = 3.86) was the most significant peak in the entire brain. At the voxel-level threshold of *p*-unc. = 0.001, no other part of the IPL was activated. Inspection of the whole-brain data confirmed the findings obtained in the main analyses that specifically targeted the rSMG subregions.

## Discussion

We found that the level of bottom-up and top-down attention allocated to stimuli during encoding affected the impact of the very same stimuli, when used as distractors, during subsequent memory retrieval. First, the distractors extracted from boundaries reduced the retrieval time more than those coming from non-boundary locations, when the temporal distance between Im1/Im2 was long in the movie. In line with our previous proposal of contingent attention capture within memory representation, such behavioural advantage was linked with increased activation in the right SMG^26^, and here more specifically localised in the PFt and PFop subregions. Second, we showed that the level of activity in the middle rSMG (a ventral part of the PF subregion) was modulated according to whether an active segmentation task was performed during encoding. The latter indicates that retrieval related activity in this part of the PF was affected by a combination of bottom-up and top-down factors of the encoding experience. These results demonstrate that the anterior and middle regions of the rSMG play different functional roles in mediating the effect of task-irrelevant distractors during temporal order retrieval, reflecting dissociable contribution of bottom-up signals generated by the external input (boundary vs. non-boundary frames) and participant’s prior task-related experiences (segmentation vs. viewing). We discuss the dissociable involvements of the anterior and middle rSMG in light of the fractionation view of the ventral parietal cortex.

The mere presence of repeated pictures can initiate episodic retrieval without having the participants to try to remember the information intentionally, indicating that retrieval processes can be incidental and stimulus-driven^43^. As the attention-to-memory model posits a role of IPL in bottom-up attentional capture to a previously experienced stimulus^25^, one may suppose that the anterior SMG activity observed here was driven by the pure stimulus-driven attention capture and/or incidental retrieval by the preferentially processed D_BORDER_ distractors^36^. Nonetheless, this is unlikely given that the D_BORDER_ distractors by themselves did not activate any of the SMG subregions significantly (and without any behavioural effect either). Instead, the effects of the D_BORDER_ distractors manifested as an interaction with the temporal distance separated by Im1/Im2 in the movie.

In long distance trials, the retrieval related activity in the anterior SMG (including the PFt and PFop, see Fig. 2) was larger - and accompanied with faster RT - when the trial included a border than a non-border distractor, indicating that any stimulus-driven capturing effects by D_BORDER_ distractors took place specifically when the two memory probes were temporally far away in the movie. We previously reported distance-dependent modulation related both to short trials (in the superior temporal sulcus) and to long trials in the supramarginal gyrus^26^, as here. In that study, we suggested that the distractor effects on long trials may relate to shifts of attention within memory representations^24^. Here, a related effect may occur, with the mnemonic status of the distractor images impacting on the retrieval of the memory probes, only when the latter involved substantial (“long”) shifts of attention between the temporal positions of the memory probes. The link between stimulus-driven attention control and episodic memory retrieval is also substantiated by previous studies that have implicated the anterior SMG in being strongly connected with the mnemonic related MTL^23^, ascribing this part of the IPL to memory based attentional processes. Here, the interaction between the effect of boundary (attention-related) and the effect of Im1/Im2 temporal distance (memory-related) supports the attention-to-memory hypothesis, showing that the bottom-up contribution, common to the segmentation and the passive viewing groups, dominated processing in the anterior part of rSMG.

Conversely, a ventral part of area PF in the middle SMG was not sensitive to the “*Distance × Boundary*” interaction when both groups were considered together. Rather, this area showed a pattern analogous to that found in the anterior SMG subregions only in the segmentation group (see Fig. 2; with a significant interaction between “*Distance*”, “*Boundary*” and “*Group*”). This showed that in this area any bottom-up effect of the distractors during retrieval was dependent on the participant’s prior task-related experiences, in line with a recent theoretical notion in the attention literature to account for the role of the IPL in mediating the joint contribution of endogenous and stimulus driven factors^31^,^32^. One possibility is that the middle SMG may encompass a higher-level processing node, together with frontal cortices (e.g., lateral prefrontal areas^28^), in a putative hierarchical network. Within this hierarchy, the higher order nodes represent the expected signal, which consists of knowledge/expectations stored in internal models determined by the top-down influences^44^. The participant’s prior task-related experiences determined how the distractors were initially encoded and accordingly, and to what extent they would influence attention allocation during retrieval. So far, evidence for such hierarchical organisations has been discussed in the context of sensory paradigms^45^ and attention to the external environment^46^. Our current results raise the possibility of extending this framework to attention capture within memory and point to the middle SMG as a possible neural correlate.

At first sight, our study seems to share some similarity with previous studies that explicitly manipulated attention at encoding^47^,^48^. These previous studies used variants of the Posner spatial cueing paradigm to direct participants attention towards (or away from) a target picture. Turk-Browne *et al.*^48^ showed that top-down attention to memory-targets can help memory formation, and associated this facilitation with activity in regions such as parahippocampal cortex. In a different study, Uncapher *et al.*^47^ showed that top-down attention to memory-targets activated the dorsal parietal cortex and improved memory success. Common to both studies was that bottom-up attention to non-targets activated the ventral parietal cortex, but diminished later memory of the targets. These studies demonstrated that different types of attention signals during encoding influence memory formation and treated attention as a scalar modifier for the memory success: more attention to the memory targets leads to better memory (see also^49^ for a review). Analogous mechanisms may have contributed to our current results. However, we further reason that memories for complex events may be formed, and stored differently into the memory engram, depending on whether these events had received processing derived from stimulus-driven attention, or a combination of both stimulus-driven and task/goal-related attention. In this sense, we consider the effects of top-down/bottom-up attention on memory encoding not to be merely “quantitative/scalar”, but rather specific and “qualitative”.

Here we propose that the task-related experience of performing the event-transition detection during encoding generated an internal construct, rendering the attended/identified distractors-stimuli a special mnemonic status (segmentation group), compared to when the very same events were not task-relevant (passive viewing group). Because of their particular status, these distractors subsequently gave rise to specific processes – and brain activation – during retrieval. In particular, it should be emphasised that our encoding material was a long dynamic movie (i.e., more complex in terms of temporal-spatial and semantic details than a single picture or scene) and that the temporal order judgement task demanded a faithful reconstruction of sequences of events for reaching correct decisions. In these settings, the memory retrieval may comprise the inclusion of rich contextual details and episodic associations, including the distractors-events. At this point, the mnemonic status of the different types of distractors would provide different levels of contextual information, modulating their impact on retrieval.

Such context-related processes may be interpreted also in the framework of other accounts of parietal activity during episodic memory retrieval. According to the output buffer hypothesis^50^, the IPL supports post-retrieval representations of the retrieved information. One specific model, the episodic buffer model, suggests that the IPL acts as a temporary storage system, supporting the maintenance and representation of the contextual details of episodic memories while a memory decision is being made^51^. In our case, the different sets of distractor stimuli – which had received different attention signals at encoding – might have provided different levels of contextual richness for the post-retrieval representation of the retrieved information, which in turn tapped into different parts of the IPL at retrieval. Other authors hypothesised that the IPL enables the working memory buffer through interactions with other cortical systems involved in memory storage, such as the MTL. In this context, the IPL retrieval activity reflects the engagement of processes that operate directly on the retrieved information, through cortical binding of relational activity (CoBRA)^52^. Based on these accounts, we attribute the different attentional signals at encoding – which resulted in different mnemonic statuses of the distractors – to causing different modulatory effects on the reconstruction-based processes during retrieval.

Concerning the parcellation of the IPL, it should be noted that schemes other than the one adopted here^10^ are also available, such as in^11^,^12^. These different schemes do not necessarily fully agree with each other, and thus the existing delineation of IPL may not be definitive (cf. Nelson *et al.*, 2010). For instance, with respect to the “*Distance × Boundary*” interaction, here we found significant peaks in two adjacent VOIs (PFt and PFop), but the whole-brain analysis showed a single cluster of activation extending across these two cytoarchitectonic regions. Also, the activation peak in area PF was located at the border between the temporo-parietal junction (TPJ) and the posterior superior temporal gyrus (pSTG)^20^, in an region that is labelled differently in the literature^3^,^11^,^53^. Some studies showed that this IPL/STG boundary region is functionally different from other parts of the IPL. For example, the pattern of lesion damage in the pSTG (but not other parts of the parietal cortex) is highly predictive of the incidence of spatial neglect^20^, suggesting that this part of the IPL may represent a convergence zone of attentional and semantic streams^19^. Other authors noted that the ventral part of the PF area often reaches to the superior temporal cortex^9^. Results based on a large sample of human brains revealed that several caudal branches of the STS ascend into the ventral segment of the IPL^54^, highlighting the importance of relating functional findings to specific gyri or sulci rather than merely ascribing the effects to an overall region. Considering that there might be smaller “sub-units” within area PF, the effects by the group-related manipulation should be interpreted more definitively with respect to the ventral IPL/STG boundary region, rather than the area PF as a whole.

We conclude that task-irrelevant stimuli presented during retrieval can influence temporal order judgment, as a function of their specific mnemonic status determined by both bottom-up and top-down influences during encoding. Task-irrelevant boundary distractors that had received processing derived from stimulus-driven attention at encoding were found to modulate the activity in the anterior rSMG (PFt and PFop subregions) during retrieval, whereas distractors that were encoded under a combination of both bottom-up and goal-related signals were found to affect retrieval activity in a ventral part of the middle rSMG. Our findings reflect a fine fractionation of parietal cortex functions, such that the SMG subregions play differing roles during memory retrieval, as a function the attentional signals allocated to the stimuli during encoding.

## Methods

### Subjects

Thirty-four participants took part in the study: 17 in the segmentation task group (6 males; *M* 24.06 years, *SD* 4.42) and 17 in the passive viewing group (6 males; *M* 24.11, *SD* 3.37). The assignment of participants to one of the two groups was randomised. The recruitment protocol ensured that none of the participants had seen any episode of the same season in which the episode in question was chosen for the experiment (see also Supplementary Information). All participants gave written informed consent, had normal or corrected vision, without known history of psychiatric/neurological problems, and were free from any MRI contraindication. The study was approved by the independent Ethics Committee of the Fondazione Santa Lucia (Scientific Institute for Research Hospitalization and Health Care), in accordance with the Declaration of Helsinki.

### Movie stimulus and event *a-priori* segmentation

The material for encoding was a 42-min episode of the American TV series “*24*” (Season 6, Episode 6, 11:00–12:00)^26^,^41^. Using a frame-by-frame approach, we coded the movie into five intertwined storylines and grouped the sequence of events into 31 clusters, with the end of each cluster representing a transition from one storyline to another. These clusters were then divided into 159 smaller epochs. The first frame of these epochs depicted a change from the preceding epoch. We defined four types of changes / epoch’s borders, as follows: (i) a story plot change from the preceding one (25 epochs), (ii) a situational change in space/locations/places within storyline (85 epochs), (iii) a natural semantic turn or development within same situational context in the same storyline (31 epochs), or (iv) a change to an epoch including non-naturalistic settings (e.g., multiple panels edited together on a single frame, 18 epochs). The epochs including non-naturalistic frames were not considered, leaving 141 epochs for image extraction for the memory probes and distractors (see also Supplementary Information). We referred to the borders separating these epochs as our set of pre-defined boundaries (“*a-priori segmentation*”), which was later validated against individual participants performance in the segmentation group (Fig. 1A).

### Encoding phase

On day 1, the segmentation group performed an event segmentation task during movie watching, whereas the viewing group watched the movie passively. We employed a between-subjects design to ensure that all participants were exposed to the same movie only once at encoding. This design avoided confounds due to any order effects of administering “segmentation task vs. viewing” treatments to the same group of subjects, thereby maximising the differences imposed by the two encoding conditions (i.e., “*Group*”).

#### Segmentation task

We employed a technique in which participants segment ongoing activity while watching the movie by pressing a button to mark boundaries defining “natural and meaningful” units^55^,^56^. Here, the participants segmented the movie by pressing a button whenever, in their judgement, one event ended and another began. All presses were recorded (*M* 140.2 key presses, *SD* 33.5) and those made within 2 s from boundaries defined by our *a-priori segmentation* were used in the extraction of boundary distractor images for the retrieval task. Consistent with the literature^57^, there was good agreement across individuals on where event boundaries are, as well as compared to our *a-priori segmentation* (number of boundaries detected: 60–98, *M* 75.8 *SD* 10.6; see Fig. 1A). Participants in the segmentation group were given an opportunity to practise the event segmentation task (see Supplementary Information).

### Retrieval phase

A total of 256 retrieval trials were presented across four fMRI-runs. Each trial included a triplet of images comprising a pair of Im1/Im2 images and a distractor image (128 D_BORDER_ and 128 D_non-BORDER_). The 2 × 2 break-down (“*Distance × Boundary*”) resulted in 64 trials per condition.

#### Images extraction

The Im1/Im2 images and D_non-BORDER_ images were identical for all participants. By contrast, the D_BORDER_ images were extracted based on event boundary judgement by the participants in the segmentation group. Subject to individual differences and subjective judgements^58^, D_BORDER_ images were determined individually according to that viewer’s responses on the segmentation task. If a participant detected a boundary matching our *a-priori segmentation* (i.e., a button press hitting on one of the *a-priori* event boundaries, see Fig. 1A), a frame would be extracted from that detected boundary for that participant. We obtained 17 individualised sets of D_BORDER_ distractor images and then assigned the participants in the viewing group to use the same sets of D_BORDER_ images. The reason for obtaining participant-specific distractor images in the segmentation group, with subsequent pairings with participants in the passive viewing group, was two-fold. First, it maximised the effect of the top-down experimental manipulation (i.e., explicit detection of boundary frames) as we ensured that—at retrieval—participants in the segmentation group were presented with events that they had explicitly reported during encoding. Second, in order to perform valid between-groups comparisons, we minimised any group differences by presenting the same stimuli/images to both groups during retrieval. The mean distance between the D_BORDER_ images and its nearest boundary were less than 1 s (*M* 0.57 s *SD* 0.26 s), whereas for the D_non-BORDER_ images it was over 10 s (*M* 12.11 s *SD* 1.16 s). The Im1/Im2 images were over 5 s from the nearest boundary (*M* 7.56 s *SD* 0.78 s). For the factor “*Distance*”, trials were assigned to the short distance conditions when the temporal distance between the two probe images was shorter than the median distance of all trials (13.4 min), otherwise to a long distance condition.

#### Temporal order judgement task

On day 2, participants received identical instructions for the retrieval task. Each trial included a triplet of static images, which were presented sequentially: First Probe Image, Distractor and Second Probe Image. The task was to choose the image that had happened earlier in the film between the two probe images. Subjects were explicitly instructed to ignore the distractor image. In this manner, the distractor images were irrelevant to the goal of the memory task. Participants indicated the target stimulus by pressing one of the two keys (Fig. 1B). The ITIs were jittered in the range of 3 – 5 s, uniformly distributed. The retrieval task was administered inside an MRI scanner, where the subjects viewed the back-projected visual stimuli via a mirror system (20 × 15° of visual angle) presented using Cogent Toolbox (http://www.vislab.ucl.ac.uk/cogent.php) running under MATLAB 7.4 (The MathWorks, Natick, MA).

### Data acquisition

All images were acquired with a Siemens Allegra (Siemens Medical Systems, Erlangen, Germany) 3 T scanner equipped for echo-planar imaging (EPI). A quadrature volume head coil was used for radio frequency transmission and reception. Head movement was minimised by mild restraint and cushioning. 32 slices of functional MR images were acquired using blood oxygenation level-dependent imaging (3 × 3 mm in-plane, 2.5 mm thick, 50% distance factor, repetition time = 2.08 s, echo time = 30 ms, flip angle = 70 degree, FOV = 192 mm, acquisition order = continuous, ascending), covering the whole cortex.

### fMRI data analysis

Data pre-processing was performed with SPM8 (Wellcome Trust Centre for Neuroimaging, London, UK; www.fil.ion.ucl.ac.uk) as implemented on MATLAB 7.4 (The MathWorks, Natick, MA). Each subject underwent four fMRI-runs (376 volumes per run). Two runs included post-boundary distractors and two included pre-boundary distractors. Since pre-boundary events do not evoke the same degree of mnemonic enhancements as for information encountered at post-event boundaries^59^, we only reported the analyses of the two runs containing post-boundary D_BORDER_ trials (see also Supplementary Information). After having discarded the first 4 volumes in each run, images were corrected for head movements. Slice-acquisition delays were corrected using the middle slice as a reference. Images were normalised to the MNI EPI template, re-sampled to 2-mm isotropic voxel size, and spatially smoothed using an isotropic Gaussian kernel of 8 mm FWHM. Time series at each voxel were high-pass filtered at 128 s and pre-whitened by means of autoregressive model AR(1).

Statistical inference was based on a random effects approach^60^, which comprised first-level analyses estimating contrasts of interest for each subject and second-level analyses for statistical inference at the group level with non-sphericity correction^61^. In the first-level, each trial was modelled as an epoch time-locked to the onset of the first probe image (duration = 5 s), convolved with the SPM8 canonical hemodynamic response (HRF) function, and with the parameters of head movements included as covariates of no-interest. For each subject, the data were best-fitted at every voxel in a multiple regression model, which considered the four trial types by crossing “*Distance*” and “*Boundary*”, plus one error-trials condition (incorrect responses or made <200 ms). For the group-level analysis, the single-subjects contrast images for the 4 experimental conditions (i.e., “boundary/non-boundary” distractors by “short/long” trials, averaged across the two fMRI-runs) were entered into a mixed design ANOVA with “*Distance*” and “*Boundary*” as within-subjects variables and “*Group*” as the between-groups variable.

#### Volume of interest (VOI)

Our analyses used five anatomical areas of interest within the right SMG (PFop, PFt, PF, PFm, and PFcm)^9^. In order to examine functionally relevant voxels within these cytoarchitectonic areas, we generated an omnibus *F*-map using the group-level ANOVA, thresholded at a low level of *p*-unc = 0.05. With this we identified voxels where the statistical model fitted the data, irrespective of the specific pattern of activation/deactivation. We presented this *F*-map in the inset at the bottom right corner of Fig. 2. The low threshold *F*-map was intersected with the five relevant anatomical areas (i.e., “anatomical areas ∩ fMRI _*F*-map_”), resulting in five VOIs for data analysis. The sizes of the VOIs were: 544 mm3 (PFop _*∩ F*-map_), 1008 mm3 (PFt _∩ *F*-map_), 5550 mm3 (PF _∩ *F*-map_), 1656 mm3 (PFm _∩ *F*-map_), and 880 mm3 (PFcm _∩ *F*-map_), see coloured patches in Fig. 2. We used the VOIs separately as five independent search volumes. Note that the VOI analyses are performed at the voxel-level, that is, data are not averaged across voxels within a given VOI. Corrected *P*-values were assigned using small volume correction^62^. Results were considered significant at *p-*svc-corr. <0.01, thus also correcting for the number of VOIs (*n* = 5).

## Additional Information

**How to cite this article**: Kwok, S. C. & Macaluso, E. Exogenous features versus prior experiences modulate different subregions of the right IPL during episodic memory retrieval. *Sci. Rep.* **5**, 11248; doi: 10.1038/srep11248 (2015).

## Supplementary Material

Supplementary Information

## Figures and Tables

**Table 1:** The table reports the results of the small volume correction (svc) analyses using the five VOIs generated by mapping the fMRI *F*-map with the cytoarchitectonic areas conjunctively [anatomical areas ∩ fMRI _*F*-map (*p*-unc < 0.05)_], see also coloured patches in Fig. 2.

VOI _∩ *F*-map_	*p-svc-corr.*	*T*	*Z*	Coordinates
“*Distance × Boundary*”				
** PFop**	**0.003**	**3.72**	**3.59**	**64–20 27**
** PFt**	**0.006**	**3.63**	**3.50**	**64–22 29**
PF	0.081	3.24	3.14	64 -24 29
PFm	0.207	2.46	2.42	64–42 37
PFcm	0.350	1.80	1.79	60–32 27
“*Distance × Boundary × Group*”: _SEGMENTATION > PASSIVE-VIEWING_				
PFop	0.152	2.17	2.14	56–24 23
PFt	0.408	1.75	1.74	68–22 29
** PF**	**0.009**	**4.02**	**3.86**	**64–38 9**
PFm	0.908	1.02	1.06	68–44 13
PFcm	0.151	2.32	2.28	58–28 19

Results were considered as statistically significant at *p-*svc-corr. < 0.01 (in **bold**), correcting for multiple comparisons for the number of VOIs. Note the dissociation between anterior SMG (PFt and PFop) and middle SMG (PF).
