# Supplementary material for: Exogenous features versus prior experiences modulate different subregions of the right IPL during episodic memory retrieval
Source: Sci Rep. 2015 Jun 9;5:11248. doi: 10.1038/srep11248 (PMC4460889; doi:10.1038/srep11248)
Supplement: Supplementary Information [file srep11248-s1.doc]

SUPPLEMENTARY INFORMATION

Exogenous features versus prior experiences

modulate different subregions of the right IPL

during episodic memory retrieval

Sze Chai Kwok* & Emiliano Macaluso

* To whom correspondence may be addressed:

Sze Chai Kwok ([sze-chai.kwok@st-hughs.oxon.org](mailto:sze-chai.kwok@st-hughs.oxon.org))

SUPPLEMENTARY METHODS

**Participants recruitment**

The recruitment protocol ensured that none of the participants had seen any episode of the same season (Season 6) in which the episode in question was chosen for the experiment. Initial screening of the participants indicated that a few subjects had seen some episodes of the previous seasons, other participants had heard about the TV series but did not see any episode, while the majority did not know anything about “*24*”. Although there was some variability of the level of familiarity with the series, we should point out that familiarity with the series seems unlikely to contribute to the retrieval task that required judging the temporal order of events in one specific (unseen) episode. Moreover, “naturalistic material” will unavoidably tap into participants’ prior knowledge and life experiences. This would imply that some of the participants were more familiar with the actors of the “*24*” series (albeit not having seen any “*24*” episode), or being followers of action-series or action-movies in general. This may contribute to the encoding of the stimuli (e.g., subjects who like action-movies might have paid more attention to the movie) and possibly to brain activity at retrieval, but, again, these uncontrolled factors appear unlikely to affect the condition-specific effects related to temporal order retrieval that were studied here.

**Encoding: Details of segmentation task practice and movie-watching setting**

The meaning of “boundary” was explained to the participants clearly. We instructed the participants that a “boundary” is defined as any abrupt or sudden change in *storyline*, *location*, or a *semantic turn* of event development. The definition excluded panorama shots (i.e., horizontal scan, movement, rotation or turning of the camera in one direction around a fixed axis) and zoom shots (i.e., a single shot taken with the effect of changing the distance between the camera and the object being filmed) [^1^](#_ENREF_1).

Participants were given several example scenarios. For instance, a spatial change from an indoor office scene to an outdoor street scene from one frame to the next constituted a transition (thus required a key press), whereas a shot jump from an actor’s face to a computer screen, and then back to the face did not. A mere change of camera perspective within the same setting did not qualify as any of the three kinds of relevant transition and did not necessitate a keypress.

Participants in the segmentation group were given an opportunity to practise the event segmentation task with five short video clips taken from an unseen season (Season 2) of the TV series. The durations of these clips were: 2′21″, 2′12″, 2′24″, 2′28″, and 2′02″. We had also carried out a frame-by-frame analysis of these clips to identify the exact locations and numbers of transitions (number of transitions: 6, 9, 8, 13, and 22 respectively). During practice, the number and location of event boundaries identified by each individual were recorded. Only button presses made within 2 s from a designated transition were qualified as hits. This 2-s threshold was strict compared to other segmentation task studies ([cf. 5-s intervals i^n 2^](#_ENREF_2)). If the participant had identified fewer than half of the total number, or more than the maximum number of the transitions determined by our frame-by-frame analysis, he/she would be reminded of the meaning of an event transition, feedback with their results, and then encouraged to repeat the practice to try to identify more (or fewer) boundaries. This practice session ensured that participants understand and familiarise the segmentation task requirement before performing the task proper for the encoding.

Once the participants had passed the practice session, the encoding session began, during which the participants performed the segmentation task while watching the movie proper. In contrast, participants in the viewing group watched the movie without having to perform the task. All participants conducted the encoding in a dimly lit room, seated ~ 60 cm away from a 19-inch computer screen (approx. 20 × 15º of visual angle). The monitor was set with a 1024 × 768 resolution and at a 60-Hz refresh rate.

**Retrieval: Frames extraction and counter-balancing**

A total of 256 retrieval trials were presented across four fMRI-runs. Each trial included a triplet of images comprising a pair of Im1/Im2 images and a distractor image (128 D_BORDER_ and 128 D_non-BORDER_). The 2 × 2 break-down (“*Distance* × *Boundary*”) resulted in 64 trials per condition. The image extraction was carried out separately for memory probe images (“Im1/Im2”), non-boundary distractor images (“D_non-BORDER_”), and boundary distractor images (“D_BORDER_”).

For the extraction of the Im1/Im2 memory probes we considered the 141 epochs identified in our *a-priori segmentation* (boundary types i, ii and iii, see main text). We extracted 256 pairs of probe images, with each image belonging to a different epoch. The images were extracted from the middle portion of the epoch, ensuring that all the probe images were at least 5 s away from any epoch boundary.

For the extraction of the 128 D_non-BORDER_ distractor images, we considered a subset of 85 epochs that included a situational change in space/locations/places within the same storyline (i.e., type ii boundaries; see also main text). For the D_non-BORDER_ distractors, the images were extracted from the middle portion of the epoch, ensuring that there were at least 10 s between the extracted distractor image and the nearest boundary. Note that, on each retrieval trial, the epoch from which the distractor image was extracted was always different from the two epochs used to extract the memory probes.

Regarding the 128 D_BORDER_ distractor images, the selection was individualised based on the subjective judgement of the participants in the segmentation group (see main text). All the D_BORDER_ images belonged to the subset of 85 epochs with type ii boundaries, and we ensured that within a retrieval trial the distractor came from a different epoch than the memory probes. To obtain the subject-specific D_BORDER_ images, we identified all the pre-defined boundaries (*a-priori segmentation*) that were also detected by that participant. From each of these borders, we extracted one or more images within 1 s from the border, making up 128 images in total. It should be noted that the D_BORDER_ images could be extracted either from *before* the boundary frames (pre-boundary, 64 trials) or from *after* the boundary frames (post-boundary, 64 trials). It has been shown that pre-boundary events do not evoke the same degree of mnemonic enhancements for episodic information encountered at post-event boundaries [^3^](#_ENREF_3), we therefore reported the analyses of the two fMRI runs containing post-boundary D_BORDER_ distractor trials. For completeness, we also analysed the pre-boundary trials. However, as expected, the pre-boundary D_BORDER_ distractors did not manifest any effect in the behavioural and fMRI analyses and were not reported further.

To avoid any systematic association between the distractor images (D_BORDER_/D_non-BORDER_) and the memory probes (Im1/Im2 images), we counter-balanced the assignment of the distractor images to specific pairs of memory probes across participants. Each pair of Im1/Im2 images was presented with a D_BORDER_ distractor in half of the subjects (i.e., “Im1 − D_BORDER_ − Im2” in 8 subjects), but with a D_non-BORDER_ distractor in the other half (i.e., “Im1 − D_non-BORDER_ − Im2” in the remaining 9 subjects). Moreover, we ensured that, in half of the trials, the memory probes were presented in the same chronological order as in the movie (i.e., “Im1-D-Im2”: with Im1 occurring before Im2 in the movie), whereas in the other half of the trials the order of presentation at retrieval was reversed with respect of the original order (i.e., again “Im1-D-Im2” at retrieval; but now with Im2 occurring before Im1 in the movie). These counter-balancing procedures ensured that any effect derived from the factor of “*Boundary*” was not confounded by any idiosyncratic relationship/timing between the memory probes and the distractor image.

**Supplementary references**

1 Smith, T. J. & Henderson, J. M. Edit blindness: The relationship between attention and global change blindness in dynamic scenes. *Journal of Eye Movement Research* **2**, 1-17 (2008).

2 Zacks, J. M., Speer, N. K. & Reynolds, J. R. Segmentation in reading and film comprehension. *Journal of Experimental Psychology: General* **138**, 307-327 (2009).

3 Ezzyat, Y. & Davachi, L. What constitutes an episode in episodic memory? *Psychological Science* **22**, 243-252, doi:10.1177/0956797610393742 (2011).
